# Fear and disgust: case report of two uncommon emotional disturbances evoked by visual disperceptions after a right temporal-insular stroke

**DOI:** 10.1186/s12883-019-1417-0

**Published:** 2019-08-13

**Authors:** Mariagiovanna Cantone, Giuseppe Lanza, Rita Bella, Giovanni Pennisi, Paola Santalucia, Placido Bramanti, Manuela Pennisi

**Affiliations:** 1 Department of Neurology, “Sant’Elia” Hospital, ASP Caltanissetta, Via Luigi Russo, 6, 93100 Caltanissetta, Italy; 2 Department of Surgery and Medical-Surgical Specialties, University of Catania, Via Santa Sofia, 78, 95125 Catania, Italy; 3 Department of Neurology IC, Oasi ResearchInstitute - IRCCS. Via Conte Ruggero, 73 - 94018 Troina, Italy; 4 Department of Medical and Surgical Sciences and Advanced Technologies Section of Neurosciences, University of Catania, Via Santa Sofia, 78, 95125 Catania, Italy; 5 IRCCS Centro Neurolesi Bonino-Pulejo, Via Provinciale Palermo, Contrada Casazza, 98124 Messina, Italy; 6 Department of Biomedical and Biotechnological Sciences, University of Catania, Via Santa Sofia, 78, 95125 Catania, Italy

**Keywords:** Emotion, Stroke, Anterior insula, Temporal pole, Neuroimaging

## Abstract

**Background:**

Emotional processes and responses are underestimated in stroke patients because the massive clinical picture of large hemispheric strokes often hides these symptoms. We report on a patient with peculiar unpleasant emotional responses after temporal stroke.

**Case presentation:**

We describe a 62-years old man with significant unpleasant emotional responses that occurred after an acute episode of confusional state, disorientation, agitation, vertigo, postural instability, vomiting, and photophobia. Since then, he complained that vision of pictures containing curved/multicolored lines or tangles was associated with an uncomfortable feeling of fear and disgust, lasting few minutes, so that he avoided looking at them. Notably, he also showed an abnormal facial expression of disgust and fear, together with neurovegetative reaction and horripilation, at the presentation of pictures of objects or animals containing curved, multicolored, or tangled lines. A post-acute infarction of the right temporal-insular region, together with mild periventricular white matter changes, were evident at the brain magnetic resonance imaging.

**Conclusions:**

The anterior insula is crucial in transforming unpleasant sensory input into visceromotor reactions and the accompanying feeling of disgust. It is also known that temporal pole modulates visceral emotional functions in response to emotionally evocative perceptual stimuli. In the present case, the ischemic lesion of anterior part of the insula and temporal pole may have caused a decoupling of emotional and visceral response to complex visual stimuli. Further reports will provide a significant contribution to the taxonomy of these complex and relatively uncommon non-motor post-stroke symptoms that negatively affect quality of life.

## Background

The insular lobe is an important somatosensory area and pain-processing center, although it also acts as a paralimbic cortex given its role in visceral sensory-motor and gustatory functions and in cardiovascular reflex regulation [1]. Intriguingly, the feeling of disgust has been linked to the insular cortex as well [2]. More recent findings indicate that even the temporal pole (TP), which is tightly connected to limbic and paralimbic regions (including the insula), has a role in both social and emotional processes. This suggests that TP may bind highly processed perceptual inputs, including complex visual stimuli, to visceral emotional processes [3].

As known, emotional responses are underestimated in acute cerebrovascular disease, as they are often hidden by other dominant clinical features of hemispheric strokes. Moreover, strokes restricted to the insular lobe and/or TP are exceptional and, therefore, the related clinical manifestations are often misdiagnosed or not recognized [1]. We describe for the first time a patient who showed significantly unpleasant feelings of fear and disgust evoked by visual disperceptions following a right temporal-insular stroke. The aim of this report was to further characterize and properly highlight these two uncommon post-stroke emotional disturbances in clinical practice.

## Case presentation

**Fig. 1:**
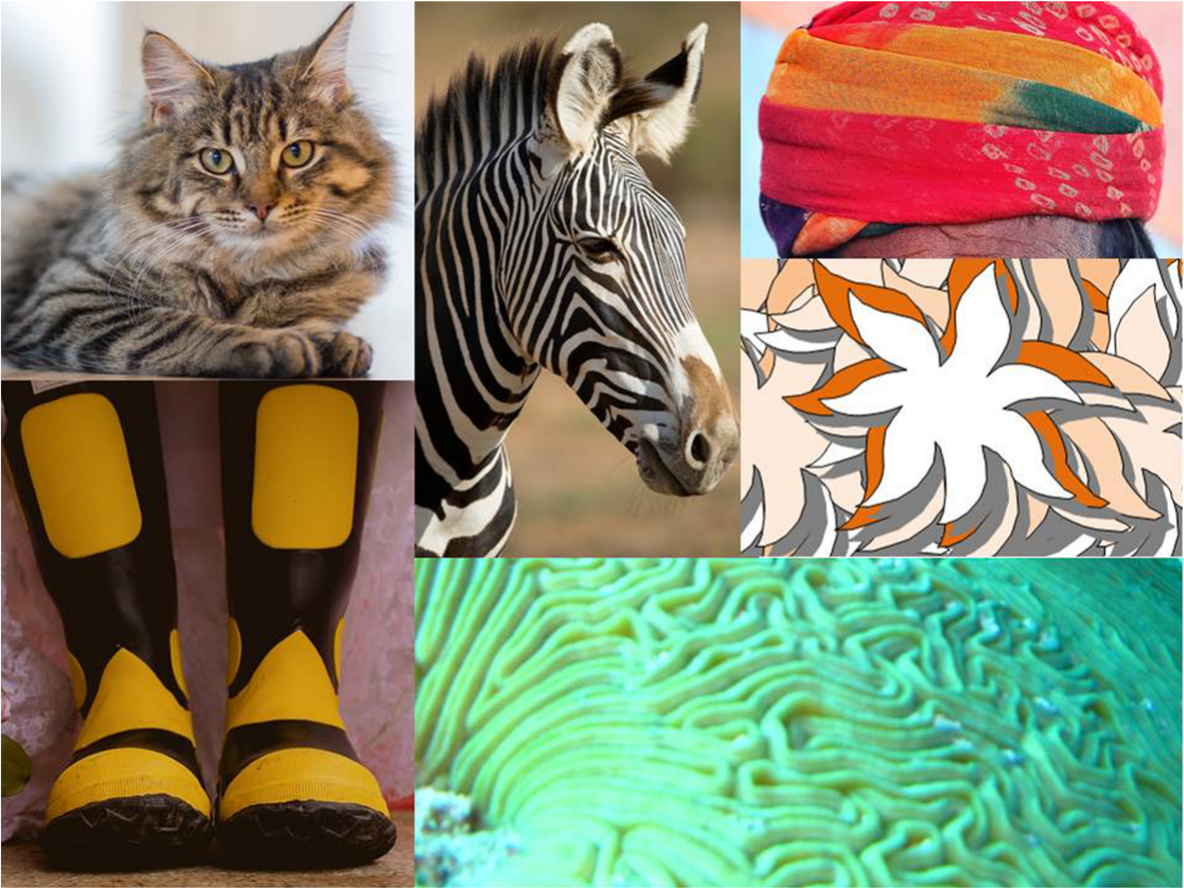
Examples of curvilinear, multicolored, or tangled images able to evoke emotions of disgust and fear in patient (acknowledged source: https://www.pexels.com)

A 62-years-old man came to our attention because of an acute episode of confusional state with disorientation, agitation, vertigo, postural instability, vomiting, and photophobia. Since then, he reported that the vision of pictures containing curved/multicolored lines or tangles was associated with an uncomfortable feeling of fear and disgust, so that he avoided looking at them. Apart from smoking, hypertension, hypercholesterolemia, and diabetes, patient’s past history was uneventful. His vascular risk factors were treated with angiotensin-converting enzyme inhibitors, statins, and oral antidiabetic drugs. There was no exposure to psychotropic drugs and no family or personal history of psychiatric disorders. The neurological examination showed diffuse hypoexcitable tendon reflexes, left sensory-motor deficit, and lack of cutaneous plantar reflex on the left side. Notably, he showed an abnormal facial expression of disgust and fear, associated with neurovegetative reaction and horripilation, lasting few minutes, at the presentation of pictures of objects or animals containing curved, multicolored, or tangled lines (Fig. 1).

**Fig. 2:**
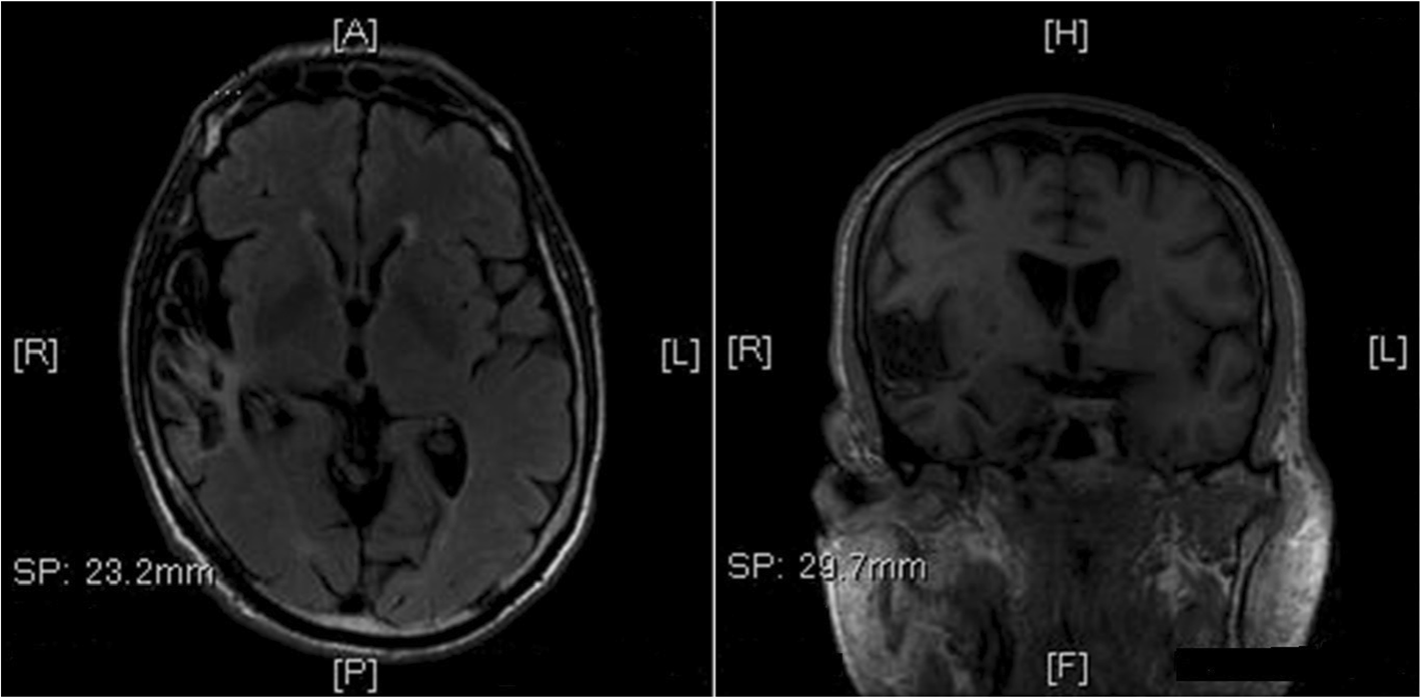
Axial FLAIR (left side) and coronal T1 3DTOF (right side) MRI images showing past right ischemic cortical-subcortical temporal-insular stroke

Blood test, ophthalmological and cardiological evaluations were normal, except for diabetic retinopathy. Neuropsychology, including mood and psychopathological assessment, showed a slight impairment of verbal and visual-spatial memory. Electroencephalography (EEG) revealed intermittent theta-activity in right frontal-temporal areas, whereas ictal EEG ruled out the possibility of an epileptic nature of these episodes. Carotid ultrasonography showed a right internal carotid artery stenosis, together with an unstable plaque. Post-acute ischemic lesion of the right temporal-insular region, together with mild periventricular white matter changes, were evident at the brain magnetic resonance imaging (MRI) (Fig. 2).

Antiplatelet therapy for secondary stroke prevention was started, along with a strict management of all vascular risk factors, towards which the patient showed good tolerability and compliance. The clinical evolution was rapidly favorable, with a substantial normalization of the neurologic examination in the 48 h following the admission. There was no adverse or unexpected event during his stay and, apart from the mentioned abnormal emotional responses; he was asymptomatic when he was dismissed.

## Discussion and conclusions

To the best of our knowledge, this is the first report of emotional sequelae evoked by visual disperceptions after a temporal-insular stroke.

As known, the insula responds to pleasant or unpleasant tastes [4], thus acting as a gustative cortex [5]. The insula is also one of the processing centers of pain and visceral sensory-motor responses. Finally, it contributes to the regulation of cardiovascular reflexes, being involved in the pathogenesis of the sudden death of neurological origin [1]. Within the insula, the anterior part (AI) is activated by both the observation of disgusted facial expressions and the emotion of disgust itself [6]. Furthermore, it has been shown that there is a specular activation of AI and frontal operculum (FO) areas of the insula and corresponding emotional experience in healthy subjects after the observation of similar reactions. Given that FO is a convergence zone where feeling states relevant for disgust are coded, lesions of FO or its connection to the insula lead to a deficit of disgust recognition. Within this frame, several studies demonstrated a crucial role of AI in transforming unpleasant sensory inputs into visceromotor reactions and the accompanying feeling of disgust [7].

On the other hand, it is also known that the TP modulates visceral functions in response to emotionally evocative stimuli. Accordingly, some positron emission tomography and functional MRI studies showed an activation of TP in response to specific emotions, such as sadness, anxiety, anger, fear, and disgust [8, 9]. TP is the most anterior part of the temporal lobe and it is closely connected with both amygdala and hypothalamus. In particular, the ventral portion of the human TP responds to complex visual stimuli, thus coupling them with visceral emotional responses. Within TP, there is also a sensory-limbic segregation of auditory, visual, and olfactory channels. For instance, surgical resection in case of tumors causes a damage of the entire TP, whereas trauma or stroke may cause a focal lesion of one sensory-limbic channel only [3]. In the present case, the ischemic lesion of AI and TP may have caused a decoupling of emotional and visceral response to complex visual stimuli.

Finally, an additional explanation might be suggested by the neurobiology underlying image perception. The observation of static images with curved, multicolored lines conveys the perception of implied motion. Accordingly, a recent study indicates that both emotion and perception of implied motion engage a similar network that includes also the activation of the insular lobe [10]. Furthermore, the observation of images with implied movement activates cognitive [11] and sensory-motor areas [12]. Thus, in this patient, the observation of images with implied movements might have triggered a dysfunctional activation of perception/emotion circuit.

In conclusion, post-stroke emotional disturbances are often underestimated and undertreated, though their relevant negative impact on health and quality of life [13]. Further reports will give a significant contribution to the taxonomy of these complex and relatively uncommon non-motor post-stroke symptoms.

## Data Availability

All data generated or analyzed during this study are included in this published article.

## Abbreviations

AI: Anterior insula
EEG: Electroencephalography
FO: Frontal operculum
MRI: Magnetic resonance imaging
TP: Temporal pole
